# The role of lymph node dissection in intrahepatic cholangiocarcinoma: a multicenter retrospective study

**DOI:** 10.1186/s12893-021-01363-4

**Published:** 2021-10-09

**Authors:** Hanjie Hu, Gang Xu, Shunda Du, Zhiwen Luo, Hong Zhao, Jianqiang Cai

**Affiliations:** 1 Hepatobiliary Surgery, National Cancer Center/National Clinical Research Center for Cancer/Cancer Hospital, Beijing, China; 2 Chinese Academy of Medical Sciences and Peking Union Medical College, Beijing, China; 3 Liver Surgery, Peking Union Medical College Hospital, Beijing, China

**Keywords:** Lymph node dissection, Intrahepatic cholangiocarcinoma, IPTW, Surgery

## Abstract

**Background:**

Lymph node dissection (LND) is of great significance in intrahepatic cholangiocarcinoma (ICC). Although the National Comprehensive Cancer Network (NCCN) guidelines recommend routine LND in ICC, the effects of LND remains controversial. This study aimed to explore the role of LND and some related issues and of in ICC.

**Methods:**

Patients were identified in two Chinese academic centers. Inverse probability of treatment weighting (IPTW) was used to reduce bias. Kaplan–Meier curves and Cox proportional hazards models were used to compare overall survival (OS) and disease-free survival (DFS).

**Results:**

Of 232 patients, 177 (76.3%) underwent LND, and 71 (40.1%) had metastatic lymph nodes. A minimum of 6 lymph nodes were dissected in 66 patients (37.3%). LND did not improve the prognosis of ICC. LNM > 3 may have worse OS and DFS than LNM 1–3, especially in the LND >  = 6 group. For patients who did not underwent LND, the adjuvant treatment group had better OS and DFS.

**Conclusions:**

The proportions of patients who underwent LND and removed >  = 6 lymph nodes were not high enough. LND has no definite predictive effect on prognosis. Patients with 4 or more LNMs may have a worse prognosis than patients with 1–3 LNMs. Adjuvant therapy may benefit patients of nLND.

## Introduction

Bile duct cell carcinoma (BCC) is a highly malignant tumor originating from the bile duct epithelium. Depending on the site, it can be classified as intrahepatic cholangiocarcinoma (ICC), hilar cholangiocarcinoma, and distal cholangiocarcinoma [1]. ICC originates above the secondary branches of the bile duct. ICC comprises about 10% of the primary malignant tumors of the liver, and is the second commonest in this group of tumors [1]. The onset of ICC is insidious. Surgery is the only effective treatment for ICC; however, only about 20% of the patients are eligible for resection at the time of diagnosis [2]. Some studies have shown that neoadjuvant therapy can depress tumors for surgery, but its role is still unclear [3]. The prognosis of ICC is poor; the median postoperative survival is about 30 months, and the 5-year survival rate is approximately 30% [4, 5]. The postoperative recurrence rate is high, and the median disease-free survival (DFS) is only 20 months. Pathogenic factors, tumor size, tumor number, lymph node metastasis (LNM), vascular infiltration, degree of differentiation, and cancer antigen (CA) 19-9 levels are related to ICC prognosis [6–12]. LNM is an important confirmed risk factor of ICC [6–8, 12].

Lymph node dissection (LND) has been used in ICC for many years. The 7th edition of the American Joint Committee on Cancer (AJCC) staging system, released in 2010 is the first independent staging system of ICC. The 8th edition of the AJCC staging system recommends the dissection of at least 6 lymph nodes in ICC for an accurate N staging [13]. LND plays an important role in determining the lymph node status in ICC and in assessing the prognosis more accurately. Many studies have focused on the relationship between prognosis and the number and location of LNMs [14, 15]. However, whether LND can improve the survival of patients with ICC remains controversial. Moreover, in AJCC8th system, the ICC N staging is divided into N0 and N1, which has some room for improvements.

From the perspective of cancer treatment, adjuvant therapy may be helpful for patients with high risk factors for recurrence. The relationship between LNM and adjuvant therapy is not fully understood. The studies of adjuvant therapy for BCC has been limited. The role of adjuvant therapy is highly controversial. In recent years, lots of the valuable results published show that adjuvant therapy does not prolong the time of relapse or survival, although they have some design limitations [16].

This study aimed to explore the role of LND and some related issues and of in ICC.

## Methods

### Data collection

Patients with pathologically confirmed ICC, who underwent a radical surgery from April 2003 to December 2018, at the Cancer Hospital and Peking Union Hospital were included in this study. The exclusion criteria included: (I) the presence of clinical or pathological distant metastases. Distant metastases do not include extraregional lymph node metastases assessed by CT and MRI because the absence of pathological evidence; (II) overall survival (OS) ≤ 1 month; (III) loss of follow-up data, which represent nothing was ever traced post-operatively; (IV) had other cancers; (V) unclear lymph node dissection state.. All experimental protocols were approved by Institutional Review Board of National Cancer Center/National Clinical Research Center for Cancer/ Cancer Hospital as it was a retrospective study. The need of informed consent was waived because it was a retrospective study. All methods were carried out in accordance with relevant guidelines and regulations.

### Statistical method

SPSS 25 (IBM, Armonk, NY, USA) and RStudio (RStudio, Vienna, Austria) were used for statistical analysis. Categorical variables were compared using the chi-square test. Inverse probability of treatment weighting (IPTW) was used to reduce the confounders of multi-center data and was implemented using RStudio. The best cutoff values were found with minimum p-value method using the X-tile software (Yale School of Medicine/Pathology/Rimm Lab, New Haven, USA)Survival analysis and verification were performed using the Kaplan–Meier method and log-rank test, respectively. Multivariate analysis was performed using the Cox proportional hazards regression model, each variable was verified to conform to the equal proportion risk hypothesis before entering the model. P values < 0.05 were considered statistically significant. All P values in this paper are derived from unilateral tests.

### The study variables

Sex, age, Hepatitis B Virus status (defined as hepatitis B surface antigen-positive), ALT, TBIL, ALB, ASA score, BMI, cirrhosis, comorbidity (Including hypertension, cardiovascular disease, diabetes, hyperthyroidism and other systemic diseases), LND (defined as at least one lymph node was removed), number of dissected lymph nodes, number of LNMs, operation time, length of postoperative hospital stay (POD time), blood loss, intraoperative blood transfusion, tumor size, carcinoembryonic antigen (CEA) levels, CA 19-9 levels, multiple disease, margin, differentiation, vascular invasion, nerve invasion, adjuvant chemotherapy, and T stage. The cutoffs for some of the variables were: X-tile: number of LNM > 3, CA 19-9 > 75 U/mL, CEA > 7 ng/mL, POD time > 9 days, operation time > 235 min, blood loss > 300 mL, tumor size > 6 cm, and age > 60 years. Some of the cutoffs: ALT, TBIL, ALB and BMI, defined in terms of standard values. ASA score ASA was divided into groups with a score of 1–2 or greater than 2. The cutoff of the number of dissected lymph nodes was 6, based on the 8^th^ edition of the AJCC cancer staging system. Adjuvant chemotherapy was defined as beginning within 3 months after surgery. The patients were divided in 2 groups: those who underwent LND (LND group) and those who did not (nLND group).

### Follow-up

The first endpoint of follow-up was the date of recurrence and the second endpoint was the date of death. Disease-free survival (DFS) and overall survival (OS) was defined from the date of the operation to the date of occurrence of endpoint events or the last follow-up time. Patients underwent first postoperative radiological examination and hematological examination 1 month after surgery. Then they review every 3 months during the first 2 postoperative years, every 6 months thereafter for 3 years, and yearly after 5 years. Follow-up was completed by telephone and searching from the data in hospital medical record system.

## Results

### Independent risk factors for ICC

#### Patient characteristics

Finally, 128 patients from Nation Cancer Center/Cancer Hospital and 104 patients from Peking Union Hospital were included in this study (N = 232). 177 (76.3%) underwent LND, and 71 (40.1%) had at least one LNM. A minimum of 6 lymph nodes were dissected in 66 patients (37.3%). There were some differences in baseline between the two groups (Table 1). POD time, operative time, intraoperative blood loss, ALT, cirrhosis rate, intraoperative blood transfusion and T stage were all higher in the LND group.

**Table 1:** Characteristics of patients with intrahepatic cholangiocarcinoma which underwent surgery (N = 251)

	LND (N = 177,76.3%)	nLND (N = 55,23.7%)	P value
*Sex*			
Female	86 (48.6)	19 (34.5)	0.68
Male	91 (51.4)	36 (65.5)	
*Age (year)*			
≤ 60	103 (58.2)	32 (58.2)	0.99
> 60	74 (41.8)	23 (41.8)	
*BMI (kg/m*^*2*^*)*			
< 25	105 (59.3)	26 (47.3)	0.12
≥ 25	72 (40.7)	29 (52.7)	
*LNM positive*			
Yes	71 (40.1)	NA	
No	106 (59.9)		
*LND number*			
< 6	111 (62.7)	NA	
≥ 6	66 (37.3)		
*LNM number*			
0	106 (60.6)	NA	
1 to 3	50 (28.6)		
> 3	19 (10.9)		
*POD time (day)*			
≤ 9	76 (43.2)	35 (63.6)	** < 0.01**
> 9	100 (56.8)	20 (36.4)	
*HbsAg + *			
Yes	32 (18.7)	9 (18.0)	0.90
No	139 (81.3)	41 (82.0)	
*CEA (ng/mL)*			
≤ 7	135 (80.4)	46 (86.8)	0.29
> 7	33 (19.6)	7 (13.2)	
*CA19-9 (U/mL)*			
≤ 75	75 (44.6)	36 (75.0)	** < 0.01**
> 75	93 (55.4)	12 (25.0)	
*ALT (U/L)*			
≤ 50	137 (77.4)	52 (94.5)	** < 0.01**
> 50	40 (22.6)	3 (5.5)	
*TBIL (μmol/L)*			
≤ 21	145 (81.9)	48 (87.3)	0.35
> 21	32 (18.1)	7 (12.7)	
*ALB (g/L)*			
< 35	18 (10.2)	2 (3.6)	0.13
≥ 35	159 (89.8)	53 (96.4)	
*ASA Score*			
≤ 2	159 (89.8)	53 (96.4)	0.13
> 2	18 (10.2)	2 (3.6)	
*Cirrhosis*			
No	28 (15.8)	16 (29.1)	**0.03**
Yes	149 (84.2)	39 (70.9)	
*Comorbidity*			
No	99 (55.9)	32 (58.2)	0.77
Yes	78 (44.1)	23 (41.8)	
*Operation time (min)*			
≤ 235	81 (45.8)	42 (76.4)	** < 0.01**
> 235	96 (54.2)	13 (23.6)	
*Blood loss (mL)*			
≤ 300	85 (48.0)	38 (69.1)	** < 0.01**
> 300	92 (52.0)	17 (30.9)	
*Intraoperative blood transfusion*			
No	106 (59.9)	42 (76.4)	**0.03**
Yes	71 (40.1)	13 (23.6)	
*Tumor size (cm)*			
≤ 6	99 (55.9)	36 (65.5)	0.21
> 6	78 (44.1)	19 (34.5)	
*Multi-disease*			
Yes	33 (18.6)	7 (12.7)	0.31
No	144 (81.4)	48 (87.3)	
*Margin + *			
Yes	22 (12.4)	2 (3.6)	0.06
No	155 (87.6)	53 (96.4)	
*Differentiation*			
Poorly	70 (39.5)	22 (40.0)	0.95
Moderately + well	107 (60.5)	33 (60)	
*Vascular invasion*			
Yes	87 (49.2)	15 (27.3)	** < 0.01**
No	90 (50.8)	40 (72.7)	
*Nerve invasion*			
Yes	60 (33.9)	7 (12.7)	** < 0.01**
No	117 (66.1)	48 (87.3)	
*T stage*			
T1	101 (57.1)	47 (85.5)	** < 0.01**
> T1	76 (42.9)	8 (14.5)	
*Adjuvant chemotherapy*			
Yes	66 (37.3)	21 (38.2)	0.90
No	111 (62.7)	34 (61.8)	

#### Prognostic factor analysis

The median follow-up time was 19 months. The total median OS was 28 months (95 confidence interval [CI] 19.025–36.975 months), and the 5-year OS rate was 32.7%. The total median DFS was 12 months, and the 5-year DFS rate was 28.0%. The 5-year OS of the nLND group and LND group was 50.6% vs. 25.7%, and the median OS was 61 vs. 21 months (95% CI 34.891- 87.109 months vs. 13.765–28.235 months, P < 0.01). The DFS of the nLND group was better than that of the LND group; the 5-year DFS was 44.2% vs. 22.3%, respectively, and the median DFS was 34 vs. 12 months, respectively (95% CI 17.612–50.388 months vs 9.039–14.961 months, P < 0.01).

LND, POD time > 9 days, CEA > 7 ng/mL, CA 19–9 > 75 U/mL, operation time > 235 min, blood loss > 300 mL, intraoperative blood transfusion, tumor size > 6 cm, multi-disease, positive margin, vascular invasion, nerve invasion, T stage > T1, ALT > 50U/L, AST > 40U/L, TBIL > 21 μmol/L, and ALB < 35 g/L were risk factors for OS in univariate analysis. These variables were included in the multivariate analysis; CA 19–9 > 75 U/mL, CEA > 7 ng/mL, positive margin, and T stage > T1 were statistically significant (Table 2).

**Table 2:** Univariate and multivariate analyses of intrahepatic cholangiocarcinoma

	OS				DFS			
	Univariate analysis		Multivariate analysis		Univariate analysis		Multivariate analysis	
	HR	P	HR	P	HR	P	HR	P
*LND*								
No	1	** < 0.01**	1	0.71	1	** < 0.01**	1	0.22
Yes	1.751 (1.157–2.650)		0.909 (0.548–1.508)		1.896 (1.259–2.857)		1.357 (0.838–2.197)	
*Sex*								
Female	1	0.91			1	0.94		
Male	1.021 (0.728–1.431)				1.013 (0.734–1.397)			
*Age (year)*								
≤ 60	1	0.44			1	0.33		
> 60	0.873 (0.619–1.230)				0.850 (0.613–1.179)			
*POD time (day)*								
≤ 9	1	** < 0.01**	1	0.24	1	**0.057**	1	0.37
> 9	2.050 (1.447–2.906)		1.299 (0.836–2.019)		1.369 (0.990–1.893)		1.215 (0.795–1.858)	
*HbsAg + *								
No	1	0.45			1	0.49		
Yes	0.839 (0.530–1.329)				1.151 (0.770–1.722)			
*CEA (ng/mL)*								
≤ 7	1	** < 0.01**	1	** < 0.01**	1	** < 0.01**	1	**0.04**
> 7	3.801 (2.526–5.718)		2.117 (1.288–3.480)		2.461 (1.623–3.731)		1.677 (1.035–2.716	
*CA199 (U/mL)*								
≤ 75	1	** < 0.01**	1	**0.03**	1	** < 0.01**	1	0.13
> 75	2.421 (1.691–3.465)		1.565 (1.044–2.346)		1.877 (1.339–2.631)		1.355 (0.918–2.001)	
*Operation time (min)*								
≤ 235	1	** < 0.01**	1	0.07	1	** < 0.01**	1	0.37
> 235	2.148 (1.523–3.029)		1.529 (0.974–2.398)		1.661 (1.200–2.299)		1.210 (0.797–1.836)	
*Blood loss (mL)*								
≤ 300	1	** < 0.01**	1	0.48	1	** < 0.01**	1	0.64
> 300	2.058 (1.465–2.891)		1.207 (0.719–2.026)		1.541 (1.116–2.126)		1.103 (0.728–1.673)	
*Intraoperative blood transform*								
No	1	** < 0.01**	1	0.37	1	0.26		
Yes	1.944 (1.381–2.735)		0.760 (0.419–1.379)		1.215 (0.869–1.699)			
*Tumor size (cm)*								
≤ 6	1	**0.03**	1	0.09	1	**0.04**	1	0.18
> 6	1.454 (1.038–2.036)		1.408 (0.949–2.088)		1.405 (1.018–1.938)		1.275 (0.893–1.819)	
*Multi-disease*								
No	1	** < 0.01**	1	0.15	1	** < 0.01**	1	0.12
Yes	2.017 (1.361–2.989)		1.467 (0.868–2.481)		1.809 (1.215–2.692)		1.507 (0.903–2.516)	
*Margin + *								
No	1	** < 0.01**	1	** < 0.01**	1	** < 0.01**	1	0.10
Yes	2.566 (1.588–4.148)		2.908 (1.642–5.152)		1.977 (1.229–3.181)		1.617 (0.916–2.855)	
*Differentiation*								
Moderately + well	1				1		1	
Poorly	1.096 (0.775–1.550)	0.61			1.438 (1.039–1.989)	**0.03**	1.583 (1.071–2.341)	**0.02**
*Vascular invasion*								
No	1	**0.01**	1	0.56	1	** < 0.01**	1	0.70
Yes	1.891 (1.350–2.650)		1.146 (0.728–1.802)		1.642 (1.189–2.267)		1.090 (0.710–1.674)	
*Nerve invasion*								
No	1	** < 0.01**	1	0.16	1	** < 0.01**	1	0.12
Yes	1.808 (1.259–2.599)		1.387 (0.877–2.194)		1.747 (1.240–2.461)		1.340 (0.874–2.054)	
*T state*								
T1	1	** < 0.01**	1	**0.03**	1	** < 0.01**	1	0.78
> T1	2.494 (1.776–3.504)		1.747 (1.071–2.848)		1.652 (1.185–2.302)		0.936 (0.594–1.476)	
*Adjuvant chemotherapy*								
No	1	0.09			1	0.78		
Yes	0.741 (0.526–1.044)				0.953 (0.683–1.331)			
*ASA*								
≤ 2	1	0.60			1	0.70		
> 2	0.849 (0.458–1.574)				1.113 (0.641–1.932)			
*BMI (kg/m*^*2*^*)*								
< 25	1	0.78			1	0.75		
≥ 25	1.050 (0.748–1.474)				0.948 (0.685–1.311)			
*Cirrhosis*								
No	1	0.14			1			
Yes	1.423 (0.893–2.268)				0.884 (0.592–1.320)			
*Comorbidity*								
No	1	0.16			1	0.99		
Yes	0.780 (0.554–1.098)				1.003 (0.726–1.384)			
*ALT (U/L)*								
≤ 50	1	** < 0.01**	1	0.91	1	0.94		
> 50	2.049 (1.379–3.045)		1.034 (0.578–1.850)		0.984 (0.634–1.526)			
*TBIL (ALB)*								
	1	**0.05**	1	0.95	1	0.54		
	1.526 (1.006–2.313)		1.020 (0.571–1.821)		0.865 (0.545–1.372)			
*ALB (g/L)*								
≥ 35	1	** < 0.01**	1	0.19	1	0.93		
< 35	2.458 (1.450–4.168)		1.628 (0.791–3.354)		0.970 (0.509–1.846)			

Bold means *P* was less than or equal to 0.05

LND, POD time > 9 d, CEA > 7 ng/mL, CA 19–9 > 75 U/mL, operation time > 235 min, blood loss > 300 mL, tumor size > 6 cm, multi-disease, positive margin, differentiation, vascular invasion, nerve invasion, and T stage > T1 were the primary factors influencing DFS. CEA and differentiation were independent risk factors in multivariate analysis (P < 0.05) (Table 2).

#### Effects of LND on prognosis

In univariate analysis, we found significant difference in survival between LND and nLND, with 5-year OS of 50.6% vs. 25.7% in nLND and LND groups. Median OS was 61 vs. 21 months (95% CI 34.891–87.109 vs. 13.765–28.235 months, P < 0.01). DFS in nLND group was better than that in LND group. 5-year DFS was 44.2% vs 22.3%, and median DFS was 34 vs 12 months (95%CI 17.612–50.388 vs 9.039–14.961 months, P < 0.01). However, in multivariate analysis, the survival difference did not exist, suggesting that the difference between nLND and LND may be due to the difference on baseline.

**Fig. 1:**
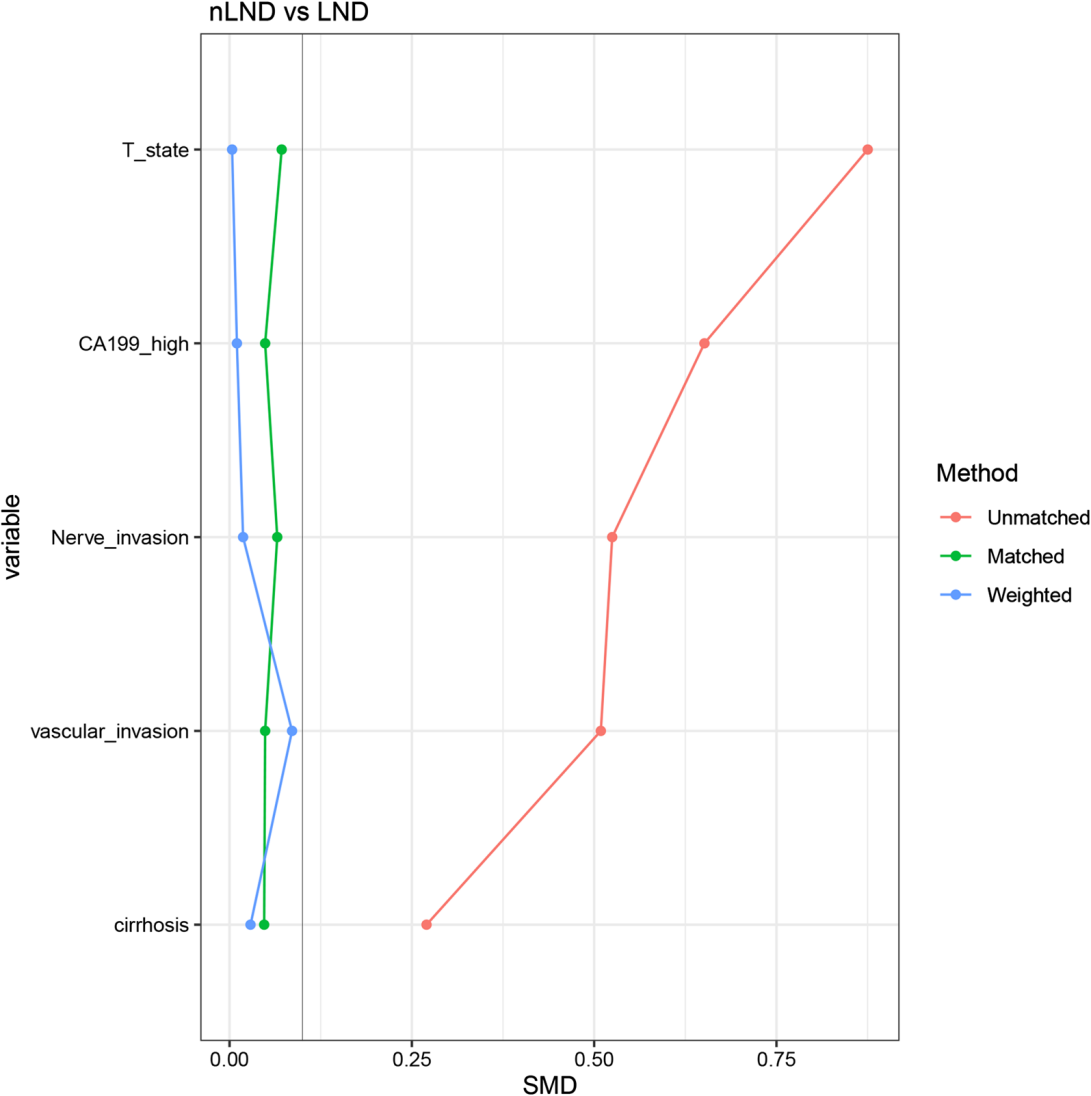
IPTW adjustments to variables between LND and nLND groups

**Fig. 2:**
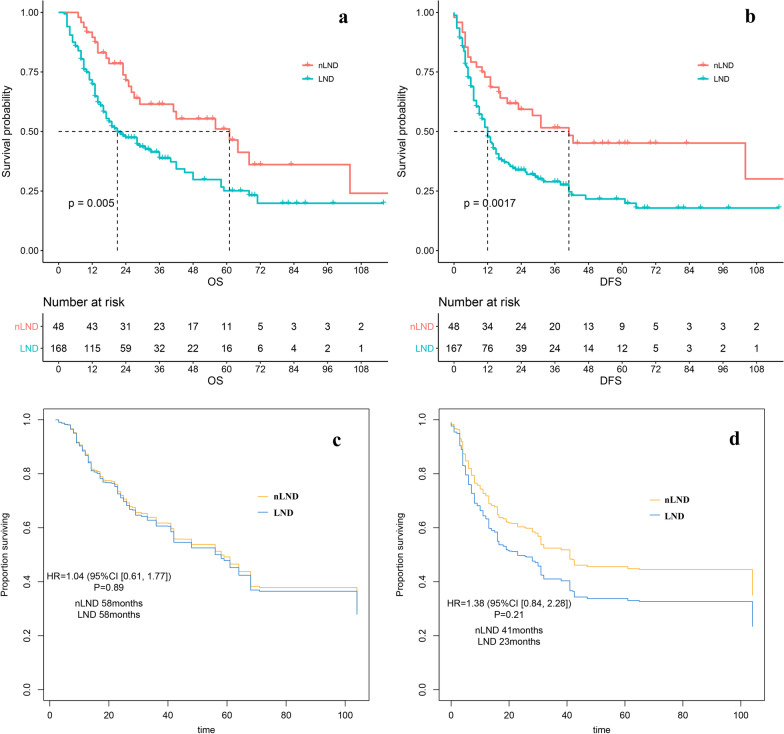
nLND vs. LND. **a** The OS without adjustment; **b** The DFS without adjustment; **c** The IPTW-adjusted OS; **d** The IPTW-adjusted DFS. There was a statistical difference in prognosis between the two groups, which was corrected after adjustment.

To further verify whether LND affected the prognosis of ICC, we used inverse probability of treatment weighting (IPTW) to reduce confounding factors to the greatest extent. We included factors that differed between groups and were likely to influence prognosis. Finally, T staging, CA199, nerve invasion, vascular invasion, and cirrhosis were included in the adjustment (Fig. 1). The standardized mean differences (SMD) showed that IPTW (weighted) effectively balanced the between-group differences. The HR of OS was 1.04(95CI 0.61–1.77, P = 0.89). The HR of DFS was 1.38 (0.84–2.28, P = 0.21). There was no statistical significance in prognosis between the two groups. (Fig. 2a–d).

### Effect of LNM on prognosis

**Fig. 3:**
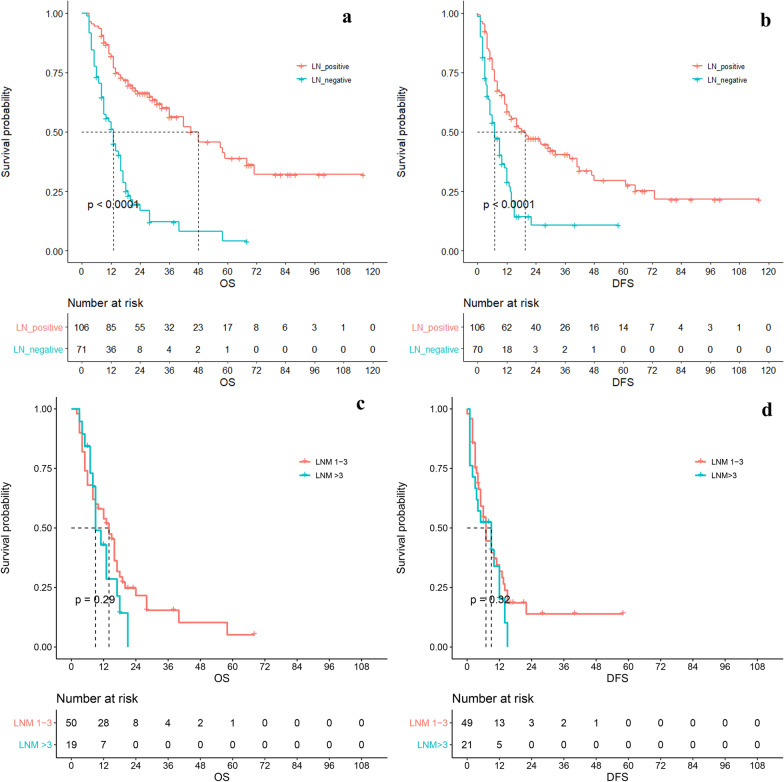
The OS (**a**) and DFS (**b**) of patients with and without LNM. The OS (**c**) and DFS (**d**) of LNM 1–3 and LNM > 3 groups

The current AJCC staging system defines the N stage in N0 (non-LNM) and N1 (at least 1 LNM), which is too simplistic. Further, we investigated the effect of LNM number on prognosis. We only conducted the study in the LND group since the situation of LNM was unknown in nLND group. The median OS was 48 months (95%CI 28.209–67.791) with a 5-year survival rate of 38.9% in LNM negative group, while the median OS was 13 months (95%CI 9.613–16.387) with a 5-year survival rate of 4.1% (P = 0.00) in LNM-positive group. The median DFS of the LNM-negative group was 19.5 months (95%CI 8.759–30.241), with a 5-year DFS of 29.6%, and the median DFS of the LNM-positive group was 7 months (95%CI 4.121–9.879), with a 5-year DFS of 0%, P = 0.00 (Fig. 3a, b). Then, the grouping was defined as LNM 1–3 (N = 52) and LNM > 3 (N = 19). The results showed that LNM > 3 had worse OS (HR 1.310 [95CI 0.657–2.612], P = 0.44). The median DFS of LNM group was 7 months, and the 2-year DFS was 10.8%. The DFS of LNM > 3 was worse, but still there was no statistical significance (HR 1.249 [95CI 0.629–2.477], P = 0.53). (Fig. 3c-d).

**Fig. 4:**
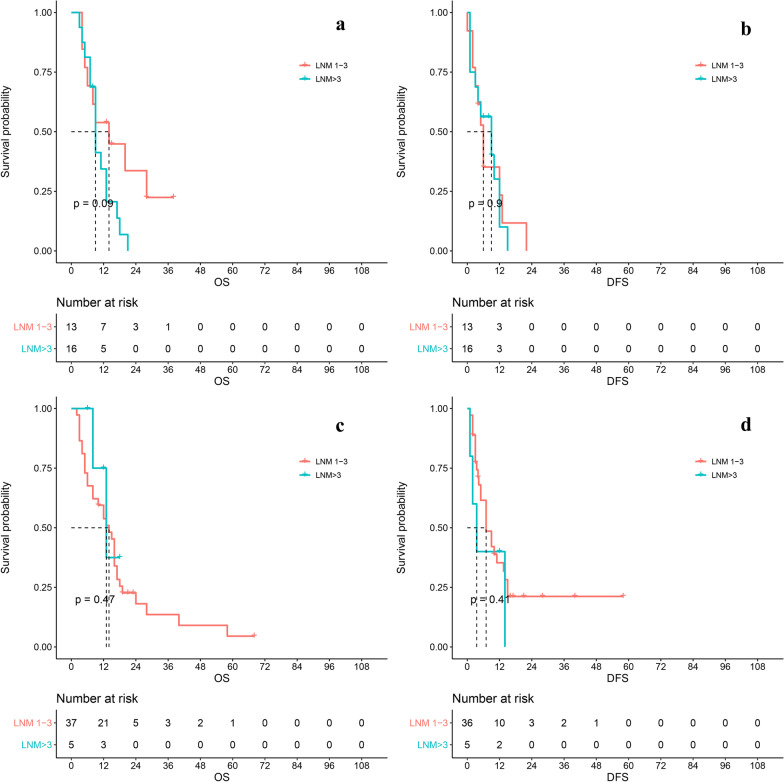
The stratified analysis on LND number. The LND > 6 group was on the top (**a**, **b**). **a** The OS of LNM number in LND > 6 group; **b** The DFS of LNM number in LND > 6 group; **c** The OS of number in LND =  < 6 group; **d** The DFS of number in LND =  < 6 group

Considering of LND number may affect the evaluation of LNM, we conducted a stratified analysis on LND > 6 or not. In LND =  < 6 group, LNM > 3 was not a prognostic risk factor (OS: HR 0.372[95CI 0.050–2.739], P = 0.33; DFS: HR 1.278[95CI 0.383–4.262], P = 0.69). In LND > 6 group, there was no difference on DFS (HR 1.249 [95CI 0.629–2.477], P = 0.9). The OS of LNM > 3 group was worse (HR 1.892 [95CI 0.805–4.447], P = 0.14), but still not statistically significant even though the P value was small. Risk table showed that all LNM > 3 patients relapsed or died within 2 years (Fig. 4a-d).

### The adjuvant therapy for nLND patients

The indications of adjuvant therapy for ICC are controversial, but the idea adjuvant chemotherapy should be performed for patients with LNM has been accepted by most doctors. The effect of adjuvant therapy for nLND patients is not clear. As we had studied above, adjuvant chemotherapy was not a independent prognostic factor in the whole cohort. 87(37.5%) patients in the cohort underwent adjuvant chemotherapy. LND and nLND groups have a similar proportion of adjuvant therapy (37.3% vs. 38.2%). In nLND group, adjuvant chemotherapy showed a protective effect on both OS (HR 0.458 [95CI 0.202–1.041, P = 0.62]) and DFS (HR 0.553 [95CI 0.250–1.222, P = 0.14]), although this was not statistically significant.

**Fig. 5:**
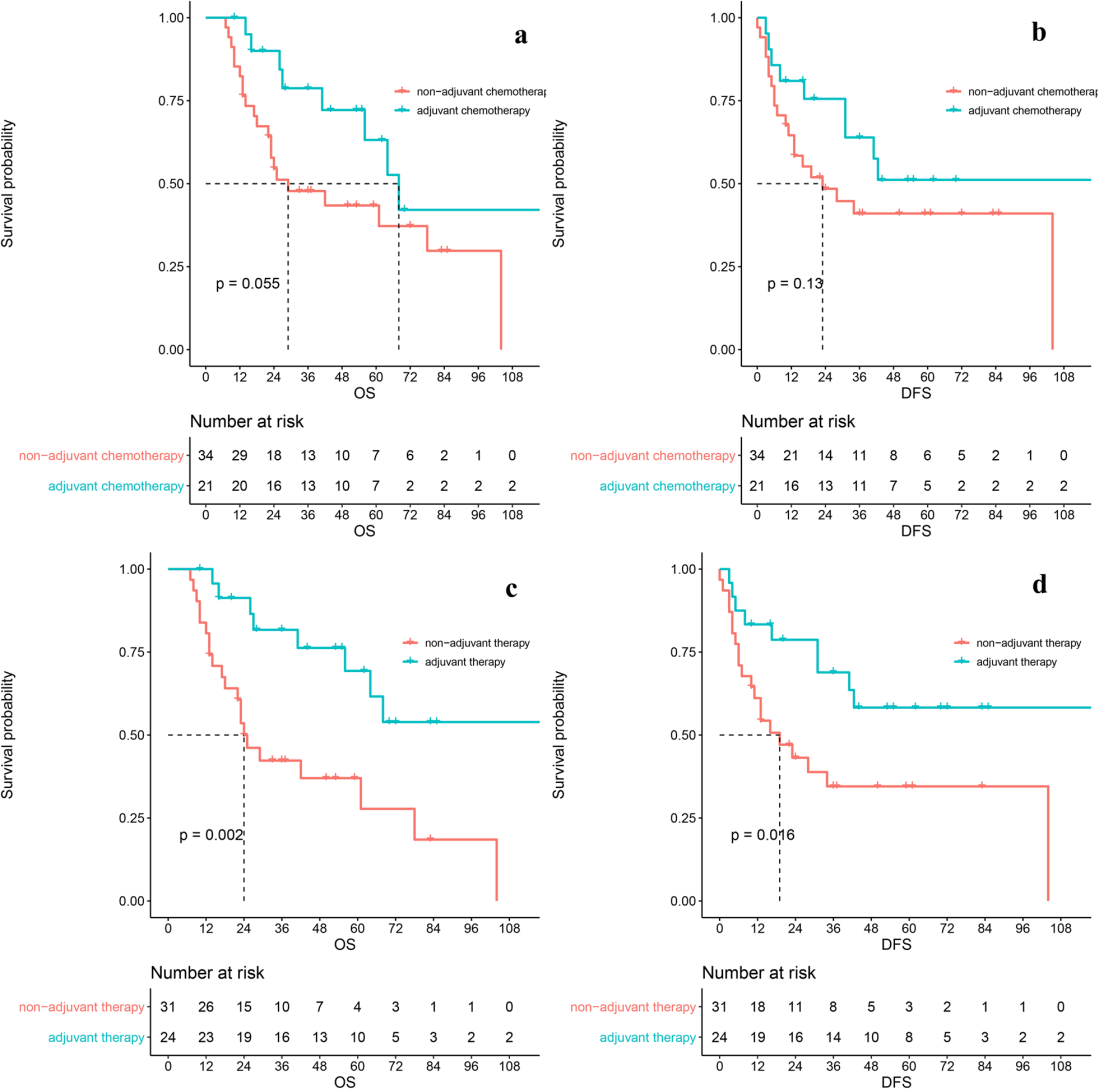
The effect of adjuvant therapy on nLND patients. **a** The OS of adjuvant chemotherapy; **b** The DFS of adjuvant chemotherapy; **c** The OS of adjuvant therapy; **d** The DFS of adjuvant therapy

Then we defined adjuvant therapy as having adjuvant chemotherapy, radiotherapy and both. 103 (44.4%) patients had adjuvant therapy. The proportion of LND and nLND groups was 44.6% vs 43.6%. In nLND group, the OS of adjuvant therapy was significant better than non-adjuvant therapy (HR 0.293 [95CI 0.128–0.669, P = 0.004]). The same conclusion applied to DFS (HR 0.391 [95CI 0.176–0.867, P = 0.02]) (Fig. 5a-d).

## Discussion

ICC is a malignant disease with a poor prognosis. The goal of surgery is complete (R0) resection. The prevalence of LNM in ICC is as high as 17–39.1% [17, 18], and LND is considered as part of R0 resection. Many surgeons believe that LND improves ICC survival. However, some researchers found that LND is only a staging operation and has little effect on prognosis [5, 19]. In our study, univariate analysis showed that the prognosis of the two groups was significantly different, and the LND group was significantly worse, which was significantly inconsistent with the general view. There were also significant differences between the two groups at baseline, which may account for this result. These can be divided into three categories: the first one, surgical factors, such as POD time, operation time, blood loss, and intraoperative blood transfusion. The second is disease factors, including CA19-9, vascular invasion, nerve invasion and T stage. The last one is patient factors, ALT and cirrhosis fall into this category. The first means the surgery is more difficult for the LND group. And the second indicates that tumors in the LND group are more advanced. Variables were well adjusted using IPTW; there was no significant difference in OS or DFS between the groups. Yoh et al. compared the effect of LND on prognosis in patients with no suspected LNM before surgery [17]. This indicated that LND improved both DFS and OS in the nLNM group. Only 112 patients were included, and some deviations in preoperative imaging assessment may exist. Ma et al. [20] found that patients who underwent extensive LND in the R0 resection group and group without distant metastases had a better prognosis, even after PSM. However, there was no difference in the whole cohort. Kim et al. obtained a result in the LND ≥ 6 and nLND groups, the former’s OS is better [21]. However, the sample size was only 68. It's important to point out that LNM status was unknown in the nLND group; therefore, all studies on LND cannot balance the LNM as a confounding factor. In our study, it could not be included as a variable in the Cox analysis and IPTW. In other words, the proportion of LNM may not same in two groups. However, LNM has a very important effect on prognosis. It also explained the importance of LND standardization. Generally, there is no high quality study that can fully prove that LND can improve the prognosis of ICC, and its significance lies in the identification of LNM. Our research supported this view, but the role of LND needs to be further explored.

The prevalence of LNM in ICC is high [22, 23]. Since LNM is a predictor of poor prognosis in ICC, LND should be performed routinely. The best approach for LND is controversial. The 8th edition of the AJCC cancer staging system suggests routine LND and removal of at least 6 LNs. This system also clearly defines regional LNs [24]. In addition to hilar nodes (common bile duct, hepatic artery, portal vein, and cystic duct nodes), regional LNs include the inferior phrenic and gastrohepatic lymph nodes in the left liver lobe. The right lobe covers the periduodenal and peripancreatic LN areas. Extraregional LNM, which was defined as distant metastases, are contraindications to surgery according to the NCCN guideline [25, 26]. The N stage may not be exact because LND is not performed or poorly done. However, some improvements in these rates have been observed. The proportion of qualified LND is increasing, though it is still not satisfactory [27]. The difference in disease factors also showed that many surgeons perform LND selectively, which is consistent with previous studies [15, 28, 29]. LND was indicated in these patients because the surgeons suspected that they would have more LNMs. In addition, as LND certainly rendered surgery more difficult. This idea was supported by the imbalances in the factors associated with surgery in our data. Caution should be taken when considering the safety of surgery. After all, LND should be performed routinely regardless of whether it improves survival. In addition, the number and range of LND should meet the requirements and be recorded in detail. The surgical details, such as whether vessels should be skeletonized, should also be further regulated.

The current N staging system of ICC is different from other BBC. In AJCC staging system, the N stage of gallbladder carcinoma and extrahepatic bile duct carcinoma were both divided into N1 (LNM 1–3) and N2 (LNM 4 or more). A cutoff of 1 (i.e. LNM-positive and negative) has been shown to effectively differentiate the patients’ prognoses [10, 12, 20, 30–33]. This conclusion was verified in our cohort. Zhang et al. put forward a new N stage model: N0 (LNM 0), N1 (LNM 1–2), and N2 (LNM > 2); they found that their model performed better in patients with at least 6 dissected lymph nodes [14]. In our study, refer to AJCC gallbladder and extrahepatic bile duct carcinoma, we grouped patients with LNM 1–3 and LNM ≥ 3. At the same time, we also analyzed the optimal LNM cutoff with X-tile software, 3 is the best, but still not statistically significant. We then tested it again in patients who met the criteria for LND (LND >  = 6), the P value was smaller (median OS, P = 0.09), but still > 0.05. In fact, all LNM > 3 patients relapsed and died within two years. This hypothesis has not been successfully tested may be due to the insufficient sample size or other bias. It also reflected that enough LND quantity can more accurately evaluate LNM.

Combining these, it can be found that the improvement of N staging and the requirements of lymph node dissection are complementary. The number of removed lymph node is prescribed primarily to reduce the false negative rate. Intraoperative frozen pathology is not routinely used in ICC surgery. If LNM has been pathologically confirmed during surgery and LND does not improve the outcome, should systematic LND still be performed? It can be speculated that some surgeons may not proceed with LND because the N stage is well defined and the risk of abdominal organ damage can be reduced. If so, we cannot further accurately study the relationship between LNM and prognosis. And this information may not be objectively recorded, which is one of the disadvantages of retrospective research. In conclusion, although this issue has been discussed for many years, more studies, especially standardized prospective studies, are still needed. Our research may bring it back into view.

Indications for adjuvant therapy in ICC are unclear. In particularly, how to make the next treatment decision when ICC patients do not undergo LND for reasons. NCCN guidelines does not mention this point, and few studies have discussed it. But the proportion of these patients is not low. Although this is not our main research area, we found some interesting phenomena. We found both similar adjuvant chemotherapy and adjuvant treatment (chemotherapy, radiotherapy, or both) rates for LND and nLND groups in our cohort, despite some adverse pathological factors in LND group. The indications of adjuvant chemotherapy and radiotherapy for ICC are not clear. Our doctors will make the adjuvant treatment plan according to the individual situation of the patient. LNM is one of the possible indications of adjuvant chemotherapy. The results from real-world showed that our treatment improved OS and DFS in patients with nLND. While this result is encouraging, it has many limitations. Firstly, we did not collect the adjuvant chemotherapy regimens and cycles of patients, so we could not conduct further analysis. In addition, reasons for adjuvant therapy were mostly not documented in patients. The number of patients treated with adjuvant radiotherapy was too small for an effective comparison, so we combined it as adjuvant therapy. Most of the early studies on adjuvant therapy had remarkable selection bias and different treatments [16]. In 2019, Results of the Prodige-12 /ACCORD-18 Phase III study was published, included all types of biliary tract tumors and found that gemcitabine plus oxaliplatin was not associated with reduced recurrence or prolonged survival [34]. BILCAP, another clinical trial, enrolled 447 patients which made it the largest trial, although only 20% of them were ICC. The result of the pre-specified ITT sensitivity analysis showed that adjuvant capecitabine extended the median OS by 17 months, and median RFS by 7 months [35]. There are still many clinical trials underway, includes chemotherapy, vEGFR inhibitors, and immune checkpoint inhibitors alone or in combination. The results are much to be expected. Although the reliability of our result was limited, we have found the possibility of postoperative adjuvant therapy benefiting patients. Further information collection and analysis will be carried out in the following studies.

This study had some limitations. Statistically, the sample size was limited, especially when subgroup analysis was performed. Data collected by different people and centers can increase bias. Patients spanned a wide range of years, during which surgical techniques and philosophies may change, and this change cannot be accurately assessed. LND was recorded only by the surgeon who performed the operation, which may make it less objective. Due to the lack of the scope and specific details of LND, we could not analyze it, and there was also a lack of unified standards for the specific operation process. Moreover, the specific plan and cycle of adjuvant therapy are not very detailed. Despite these, we hope that this study can provide a reference for ICC surgery, prognostic model, staging system, and adjuvant therapy for subsequent studies.

## Conclusions

CA 19-9, CEA, operative time, positive surgical margin, and T stage were independent risk factors for OS; CEA and differentiation were independent risk factors for DFS. The proportions of patients who underwent LND and removed >  = 6 lymph nodes were not high enough. LND has no definite predictive effect on prognosis. Patients with 4 or more LNMs may have a worse prognosis than patients with 1–3 LNMs. Adjuvant therapy may benefit patients of nLND.

## Data Availability

The datasets used and/or analysed during the current study are available from the corresponding author on reasonable request.

## Abbreviations

LND: Lymph node dissection
nLND: Not undergo LND
BCC: Bile duct cell carcinoma
AJCC: American Joint Committee on Cancer
ICC: Intrahepatic cholangiocarcinoma
NCCN: National Comprehensive Cancer Network
PSM: Propensity score matching
IPTW: Inverse probability of treatment weighting
LNM: Lymph node metastasis
POD time: Postoperative hospital stay
OS: Overall survival
DFS: Disease-free survival
